# Antiretroviral medication treatment for all HIV-infected individuals: a protocol using innovative multilevel methodologies to evaluate New York City's universal ART policy among problem substance users

**DOI:** 10.1186/s12913-016-1554-8

**Published:** 2016-08-02

**Authors:** Aimee N. C. Campbell, Don Des Jarlais, Cooper Hannah, Sarah Braunstein, Susan Tross, Laura Kersanske, Christine Borges, Martina Pavlicova, Kevin Jefferson, Howard Newville, Laurel Weaver, Margaret Wolff

**Affiliations:** 1 Department of Psychiatry, Columbia University Medical Center, New York State Psychiatric Institute, 1051 Riverside Drive, Box 120, New York, NY 10032 USA; 2 Icahn School of Medicine at Mount Sinai, 39 Broadway, 5th Floor, New York, NY 10006 USA; 3 Department of Behavioral Science and Health Education, Emory University, Rollins School of Public Health, 1518 Clifton Road NE, Room 568, Atlanta, GA 30322 USA; 4 New York City Department of Health and Mental Hygiene, 42-09 28th Street, Long Island City, NY 11101 USA; 5 Department of Psychiatry and Behavioral Health, Mount Sinai St. Luke’s Hospital, 1111 Amsterdam Avenue, 11th Floor, New York, NY 10025 USA; 6 Department of Biostatistics, Mailman School of Public Health, Columbia University, 722 West 168th Street, 6th Floor, #637, New York, NY 10032 USA

**Keywords:** HIV/AIDS, Adherence, Antiretroviral therapy, Substance use, Affordable care act

## Abstract

**Background:**

The intersection of HIV-related health outcomes and problem substance use has been well documented. New York City continues to be a focal point of the U.S. HIV epidemic. In 2011, the NYC Department of Health and Mental Hygiene (NYC DOHMH) issued a recommendation that all HIV infected individuals should be offered antiretroviral therapy (ART) regardless of CD4 cell count or other indicators of disease progression. This policy is based in the concept of “treatment as prevention,” in which providing ART to people living with HIV (PLWH) greatly reduces the likelihood of HIV transmission, while also improving individual health. The “ART for ALL” (AFA) study was designed to inform modifications to and identify gaps in the implementation of universal ART, and specifically to help guide allocation of resources to obtain local policy goals for increasing viral suppression among PLWH who have problem substance use.

**Methods/Design:**

The AFA Study is informed by two complementary frameworks: Glasgow and colleagues’ RE-AIM model, a multi-level framework developed to guide the evaluation of implementation of new policies, and Bronfrenbrenner’s ecological systems model, which conceptualizes the bi-directional interplay between people and their environment. Using multi-level data and mixed methods, the primary aims of the AFA Study are to assess rates of viral load suppression, using the NYC HIV Surveillance Registry, within 12 months of HIV diagnosis with (a) yearly cohorts of high-risk-to-transmit, difficult-to-treat, substance using patients recruited from NYC Sexually Transmitted Disease clinics and a large detoxification unit and (b) yearly cohorts of all newly HIV diagnosed people in NYC. Further goals include (c) recruiting cross-sectional samples of HIV/AIDS service providers to assess ART initiation with problem substance users and d) examining geographic factors that influence rates of viral load suppression. An Implementation Collaborative Board meets regularly to guide study procedures and interpret results.

**Discussion:**

The AFA Study has the unique strength of accessing and analyzing data at multiple levels using mixed methodology, taking advantage of NYC DOHMH biomedical surveillance data. If successful, others may benefit from lessons learned to inform local and state policies to improve the health of PLWH and further reduce HIV transmission.

## Background

The intersection of HIV-related health outcomes, such as HIV transmission and substance abuse, has been well documented. Problem substance is indicated in both HIV prevention and treatment strategies. Beyond the risk of increased rates of HIV transmission due to injection-related behaviors (e.g., sharing needles), sex under the influence of drugs and alcohol is frequently associated with HIV transmission [1–6]. Persons with problem substance use are also less likely to engage in treatment for HIV infection [7–9], to be retained in treatment [10], and to adhere to antiretroviral treatment (ART) schedules [11, 12]. It is also well established that substance users are at higher risk for sexually transmitted infections (STI) [4, 13–15]. Prevalence of recent substance use in STI clinic samples include up to 50 % reporting heavy alcohol use in the past 30 days and 47 % with illicit substance use in the past year [16]. Sexually transmitted infections biologically facilitate HIV transmission [17–19], and diagnosis of a sexually transmitted infection is an indicator of risky sexual behavior. Thus, ART initiation programs should target and respond to the needs of substance users [20].

### Shifting context of the NYC HIV epidemic

New York City continues to be a focal point of the U.S. HIV epidemic; however, significant shifts in the epidemic are underway. In 2010, there were 3353 new HIV infections in NYC [21]. As in the United States more broadly, HIV infection in NYC is heavily concentrated in racial/ethnic minority communities [10, 22]. In 2010, African American/Black and Hispanic/Latino individuals made up 47 and 31 %, respectively, of new HIV diagnoses in NYC [21], while comprising 23 and 29 %, respectively, of the NYC population [23]. Since 2010, there have been continued decreases in the number of HIV diagnoses in NYC. In 2013, there were a total of 2832 cases of new HIV diagnoses in NYC, a reduction from 2990 in 2012 and 3225 in 2011 [21].

Viral load suppression within 12 months of diagnosis has also been increasing over time. Data from 2006 to 2009 and published in 2013 indicate that achieving viral suppression (≤200 copies/mL) within 12 months of diagnosis was 39.8 % (36 % in 2006 and 45 % in 2009) [24]. More recently, according to data from 2013, viral suppression within 12 months was 69 % [25]. This proportion was moderated by age (younger people have lower rates of suppression) and CD4 count at diagnosis (those with greater than 500 cells/mm3 were less likely to be suppressed) [25]. Durable viral suppression (DVS), a marker of high engagement in care, has also shown similar increases over the last decade. Only 38.6 % of people living with HIV (PLWH) reached DVS in 2006–2007, with DVS defined as all viral load tests being ≤200 copies/mL over the 2 year period among people with 2 or more viral load tests [26]. In 2010–2011, the proportion with DVS had increased to 52 % - an overall increase of 37 % from 2006–07 to 2010–11 [27].

Longitudinal data from a recent analysis of persons who inject drugs, recruited from a large detoxification unit and methadone maintenance program in NYC, also demonstrated that the percentage of persons at risk for transmitting HIV (defined as those HIV positive and engaged in distributive needle sharing) decreased from 21 % in 1990–1994 to 2 % in 2007–2014 [28]. These promising trajectories suggest a shift in the HIV epidemic among persons who inject drugs.

Despite these improvements, much work remains, especially among Black and Latino young men who have sex with men (MSM) and individuals with problem substance use. To change the HIV landscape in a lasting way at least 80 % of PLWH must be virally suppressed [25, 29]. It is likely that additional innovative practices, guidelines, and policies will be needed to increase the proportion of PLWH who reach viral suppression within 12 months of diagnosis.

### Universal antiretroviral therapy: treatment as prevention

In December 2011, the New York City Department of Health and Mental Hygiene (NYC DOHMH) issued a recommendation that all HIV infected individuals should be offered ART regardless of CD4 cell count or other indicators of disease progression [30]. This policy is based in the concept of “treatment as prevention,” in which providing ART to HIV seropositives greatly reduces the likelihood of HIV transmission [31–34], while at the same time improving individual health outcomes [35–37]. In combination with other HIV prevention programs in New York (e.g., Seek, Test, Treat, and Retain [20, 33, 38, 39], free condom distribution, syringe exchange [40, 41], opioid agonist treatment [42, 43], and most recently pre-exposure prophylaxis [PrEP] for high-risk individuals)—the universal ART policy aims to improve outcomes along the HIV care cascade [44] and help to facilitate realization of an AIDS-free generation.

NYC’s implementation of universal HIV treatment has been gradual. Following the initial release of early treatment recommendation in December 2011, there were accompanying press coverage and FAQ’s for providers and consumers, revisions to the early treatment brochure distributed to testing and Ryan White funded clients (under- or uninsured clients who receive federal funding for HIV health care delivery), and the development of a texting campaign for young MSM to encourage early treatment. Universal ART was added to pre-existing efforts such as the 2010 New York State law that health providers of all types offer HIV testing to consumers aged 13–64 (and younger and older patients at high risk) at all visits [45]. Multi-level, borough-wide campaigns using mass media, provider training, community outreach, and other methods, urged HIV testing [46]. In 2013, the NYC DOHMH Bureau of Sexually Transmitted Disease (STD) Control assessed their HIV primary care referrals to ensure providers were implementing new recommendations and, specifically that referrals were made only to providers using universal ART guidelines. A primary initiative that has recently been implemented by the NYC DOHMH is to provide data on viral load and linkage to care to Health and Hospitals Corporation and other high-volume HIV care providers. Reporting of viral load outcomes will be a critical feedback loop to providers for increasing awareness of their patients’ outcomes and working to achieve higher suppression rates.

Placing larger numbers of HIV diagnosed patients on ART and providing support services so that they achieve viral suppression will undoubtedly require additional resources. The new policy will be incorporated into new contracts between NYC DOHMH and AIDS service providers for use of Ryan White funds. These new contracts will require that services be billed to Medicaid when possible, so that Ryan White funds may be used for those not Medicaid eligible (and who do not have private health insurance).

### Affordable care act legislation

Concurrent implementation of Patient Protection and Affordable Care Act [47] legislation will also be an important factor in the evaluation of universal ART among PLWH in NYC. The NYC DOHMH is in the process of planning how to respond to the potential for the Affordable Care Act to alter the landscape of HIV treatment and care, especially through Medicaid expansion and its integration with state-based insurance exchanges. For example, since Ryan White funding has a payment of last resort requirement, Ryan White funded services will need to adjust to the current implementation of Medicaid Health Homes, including income eligibility threshold changes for Medicaid recipients and the implementation of Health Insurance Exchanges.

### An opportunity to evaluate the “ART for ALL” policy: AFA study

In the context of a shifting HIV epidemic in NYC and the overall changes across the healthcare continuum, the “ART for ALL” (AFA) Study was designed to not only provide critical information that could be used to help achieve policy goals for increasing viral suppression among PLWH, but to remain flexible within the changing context of the NYC HIV epidemic. The AFA Study has the unique strength of accessing and analyzing data at multiple levels, with mixed methods. It also has the advantage of integrating NYC DOHMH biomedical surveillance data, both with individual behavioral assessments and geospatial (i.e., neighborhood) variables, to provide information about potential moderators and mediators of HIV treatment outcomes. Data collected from the AFA Study will be used to inform modifications to or identify gaps in the implementation of universal ART, and in particular, to help guide allocation of resources to obtain local policy goals. The research will be conducted with the NYC DOHMH as a full partner and includes a Collaborative Board with service providers, clinical researchers, and consumers to review data and make formal recommendations. If successful, there is potential that others may benefit from lessons learned to improve the health of people with HIV and reduce further HIV transmission – leading the way towards an AIDS-free generation.

**Fig. 1:**
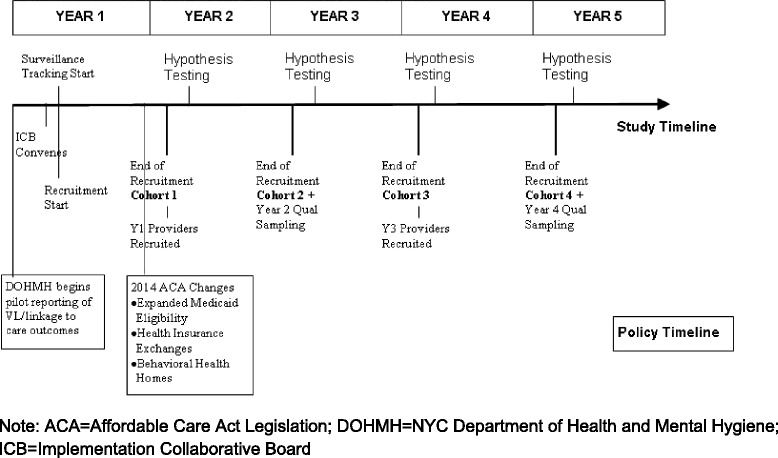
AFA Study Implementation Timeline

The primary aims of the AFA Study are to assess rates of viral load suppression within 12 months of diagnosis with (a) yearly cohorts of high risk to transmit, difficult to treat, substance using patients and (b) yearly cohorts of all newly HIV diagnosed people in NYC. Further goals include (c) recruiting cross-sectional samples of HIV/AIDS service providers to assess, via quantitative and qualitative methods, ART initiation with problem substance users and organizational factors which facilitate or impede ART initiation; and d) examining geographic factors (i.e., United Hospital Fund (UHF) level data) that influence rates of viral load suppression. NYC DOHMH HIV surveillance data allow for the tracking of key clinical outcomes (e.g., via viral load and CD4 count data). The study is intended to inform implementation of the universal ART policy, as well as treatment changes brought about by the full implementation of the Affordable Care Act, and guide allocation of resources to reach this policy goal (see Fig. 1). As such, the AFA Study was purposefully designed to be flexible and adjust to the changing HIV landscape in NYC. The design, especially eliciting ongoing Implementation Collaborative Board feedback, also allows for modification of sampling and methods.

## Methods/Design

### Conceptual framework

**Fig. 2:**
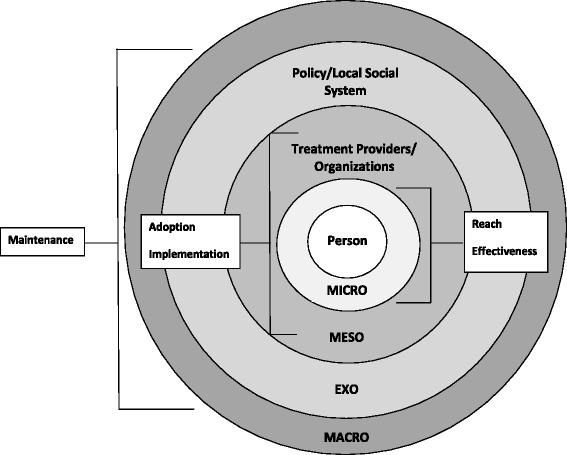
Ecological Systems and RE-AIM Conceptual Model

The AFA Study is informed by two complementary frameworks: the RE-AIM [48] and ecological systems [49] models (see Fig. 2). The RE-AIM model [48] is a multi-level framework developed to guide the evaluation of implementation of new policies. RE-AIM is comprised of five essential dimensions: 1) Reach – measure of percentage (and characteristics) of people who are affected by the policy; 2) Efficacy – positive and negative individual behavioral, quality of life, satisfaction, and physiologic outcomes of the policy; 3) Adoption – proportion and representativeness of participating and non-participating providers/settings; 4) Implementation – extent to which the policy or intervention is delivered as intended, including participant adherence and provider fidelity; and 5) Maintenance – long-term participant outcomes and the extent to which policies become part of routine practice.

In this study, the RE-AIM model is utilized within the context of the ecological systems model [49], which conceptualizes the bi-directional interplay between people and their environment. The ecological systems model is useful for explaining syndemic health disparities associated with HIV (e.g., substance abuse, mental health, sexually transmitted infections [50, 51] and specifically how multi-level factors influence individual HIV treatment linkage, retention, and ultimately viral load suppression. Micro-level systems include the individual and his/her immediate interpersonal interaction within family or social supports, school, work, and services. “Proximal processes”, or regular reciprocal interactions within an individual’s immediate environment that vary in duration, frequency, interruption, timing, and intensity [52–54], are an important part of the microsystem. The mesosystem is comprised of interactions between microsystem settings. Provider and organizational settings fall within the mesosystem (e.g., provider knowledge). The exosystem includes dimensions of the larger social system in which the individual is embedded, but over which he or she has limited control. Place characteristics (e.g., poverty rates, the epidemiologic environment, such as HIV prevalence, and the health service environment, such as spatial access to healthcare) are aspects of the exosystem. The macrosystem covers overarching “prototypical” culture and structures (e.g., economic systems, social norms), often implicit in social practice [55]. In the proposed study, micro, meso, exo and macro system environments will be evaluated among clients, providers, and the broader HIV treatment system, including Affordable Care Act legislation.

### Micro-system: STD/detox cohort and NYC surveillance cohorts

Individual-level data will be obtained from two separate cohorts: (1) STD/Detox and (2) HIV surveillance (i.e., all new HIV diagnoses in NYC). The STD/Detox cohort allows for the collection of comprehensive data from a subgroup of HIV-infected individuals, while the HIV surveillance data allows for examination of the entire population of newly HIV-infected individuals during the study.

#### STD/detox cohort

The STD/Detox Cohort, as the name implies, will be recruited from (1) NYC DOHMH STD clinics, primarily from the two largest clinics located in Manhattan and Brooklyn, and (2) the 139-bed inpatient general drug detoxification unit at Mount Sinai Beth Israel Medical Center in Manhattan. Eligible participants 1) have a new HIV diagnosis or have been previously diagnosed but have been out of care in the last year (i.e., not seen by an HIV primary care provider), 2) report problem substance use, and 3) are able to speak and understand English. HIV diagnoses will be confirmed through a match of study data with the NYC DOHMH HIV surveillance registry. Problem substance use is broadly defined to ensure range of severity: injection drug use or injection drug use combined with male to male HIV sexual transmission risk; illicit drug use in the prior 12 months, including club drugs; or heavy drinking (i.e., more than four drinks for men and more than three drinks for women on one occasion, following NIAAA guidelines [56]) in the past 30 days.

Recruitment locations were selected because these patients are likely to be at high-risk of transmitting HIV to others (i.e., high rates of problem substance use [16] and actively engaged in unsafe sexual behaviors) and they will provide representation of a spectrum of substance use problems – i.e. from more severe substance use disorders on the detox unit to variable severity at the STD clinics. Demographic profiles using prior research and STD electronic medical record data from 2012 suggest that 90 % of participants will be male; 55 % of STD clients will be 29 years of age or younger, while the mean age of detox clients will be 34 years and 35 % will identify as Hispanic, 31 % Black, and 27 % White [57]. In prior research, younger HIV seropositive individuals have demonstrated greater delay in treatment linkage [58].

Four yearly cohorts of patients are being recruited (2014–2017; target *N* = 300, 75 per cohort). It is expected that approximately 200 patients will be recruited from STD clinics and 100 from detox. In 2011, 447 clients received a positive test for HIV in NYC STD Clinics; 244 were from the two largest clinics (55 %). Approximately 4500 clients are seen in the Beth Israel Detox Program each year; 10 % are HIV positive [28].

At first contact with the client, research staff will provide a brief description of the study and ask if the client is willing to provide contact information. The research assistant will contact interested clients by telephone and will obtain the client’s verbal consent for a short screening interview. The screen will determine eligibility and the following information will be collected: age, sex, gender, race/ethnicity, transmission risk category, previous HIV testing, and assessment of problem substance use. Following screening, eligible and interested clients provide written informed consent and are formally enrolled in the study. All enrolled participants complete a baseline assessment and four follow-up assessments at 6-month intervals over the next two years. All measures will be administered at all assessment time points (except for static demographic characteristics). All assessment visits will take about 90 min. This study was approved by the Mount Sinai St. Luke’s/Roosevelt Institutional Review Board (IRB), Mount Sinai Beth Israel IRB, and the NYC DOHMH IRB. Table 1 details domains, variables, and measurement tools across data sources based on the RE-AIM dimensions. For patient-level data collection, the dimensions Reach and Efficacy will be assessed. Maintenance will be assessed using patient follow-up data.

**Table 1:** Measurement domains, variables, and data source by Ecological System and RE-AIM outcomes for patient, provider and geographic analyses

Indicator/domain	Variable(s)	Measurement source
Patient level: Person (MICRO): Reach, Efficacy, Maintenance		
Demographic Characteristics	Age, race/ethnicity, sex, gender, marital status, living situation, dependents, education and income, insurance, legal, immigration status, religion and spirituality, medical history	AFA Survey
Primary Outcome: Viral load suppression	Durable Suppression: two consecutive tests with ≤ 200 HIV-1 RNA copies per mL of plasma	HIV Surveillance Registry
	Viral Suppression: most recent test with ≤ 200 HIV-1 RNA copies per mL of plasma	
Secondary Outcome: Linkage	Occurrence of first CD4/VL test date after study enrollment	HIV Surveillance Registry
Secondary Outcome: ART Initiation	ART regimen initiated	AFA Survey
Secondary Outcome: Treatment retention	Sustained engagement or regular care, ≥ 1 primary care visit in each 6 month interval	HIV Surveillance Registry
Secondary Outcome: HIV medication (ART) Adherence	Adherent at threshold (≥90 %) for all ART medications	Visual Analogue Scale [72–74]
Secondary Outcome: Quality of Life	Health-related quality of life	EQ-5D EuroQoL [75]
Secondary Outcome: Sexual and Drug Use Risk	Sexual partners, unprotected sex acts, needle/works sharing	Modified Risk Assessment Battery and Risk Behavior Scale [76, 77]
Predictor: Substance Use	Illicit drug use (days), alcohol (days, # drinks), substance use disorder severity	TLFB [78]; QuickTox 8-panel Drug Screen Dipcard; Fagerstrom Test for Nicotine Dependence [79]; DSM-V Checklist for Substance Use Disorders [80]
Predictor: Mental Health	Psychological distress	Kessler10 [81], Personality Inventory for DSM-V Brief Form [82]; Posttraumatic Stress Symptom Screener [80]
Predictor: Social Support	Social network size/quality; disclosure of HIV to family/friends	MOS Social Support Survey [83]; HIV Disclosure Status Questionnaire [84]
Predictor: Service Utilization	Medical, substance abuse treatment, mental health, case management, Ryan White-funded services	Modified Treatment Services Review [85]
Predictor: Attitudes	Attitudes towards primary HIV provider professional	Attitudes Towards HIV Health Care Providers Scale [86]; Medical Mistrust Index [87]
Predictor: ART Barriers	Barriers to ART adherence	Adherence Barriers Questionnaire [88]
Predictor: Stigma	Experiences and perceived discrimination and stigma	MIDUS Daily Discrimination Scale [89]; Social Impact Scale [90]
Predictor: Knowledge	HIV treatment knowledge	HIV Treatment Related Knowledge Inventory [91, 92]
PROVIDER/PRIMARY CARE LEVEL (MESO): Adoption, Implementation, Maintenance		
Professional Characteristics	Age, sex, race/ethnicity, education, years since terminal degree, HIV care (years in current position/providing HIV care, caseload)	AFA Survey
Predictor: Organizational characteristics	Location, service type, size, staffing, service flexibility, impact of ACA, staff attributes, organizational culture and climate	Perceived Environmental Uncertainty (unpublished measure, D’Aunno, 2013); Change Survey [93, 94]; Survey of Organizational Attributes for Primary Care [95]; Medical Group Practice Culture [96]
Predictor: Knowledge	Knowledge of ART guidelines; perceived self-efficacy to follow ART guidelines	AFA Survey
Predictor: Attitudes	Attitudes and ideology toward patients and the provider/patient relationship, including patients with substance use problems	Attitudes About Patients with Substance Use Disorders [97, 98]; Patient-Practitioner Orientation Scale [99]
NEIGHBORHOOD/GEOGRAPHIC LEVEL (EXO): Reach, Efficacy, Maintenance		
Predictor: Socioeconomic Conditions	Poverty rate; median income; unemployment rate; educational attainment; percent of residents who are non-Hispanic White	2011 American Community Survey, 5-year estimate [100]
Predictor: Health Service	Spatial access to HIV primary care	2013 Data pull from HIVMA, AAHIVM, NYSDOH AIDS Institute Provider Database
Predictor: Social Disorder	Off-premises alcohol outlet density; vacant housing	2009–2012 U.S. Census Bureau’s Zip Code Business Patterns [101–103]; 2009–2010, 2012 USPS Delivery Stats Product [104–106]
Predictor: Social Cohesion	% of housing units leased; % households that moved into the neighborhood in last year	2011 American Community Survey, 5-year estimate [107]

#### NYC HIV surveillance cohort

The HIV Surveillance data will serve as the source of data for the STD/Detox patient-level outcomes, as well as be used to create yearly NYC surveillance cohorts (i.e., all new HIV positive individuals in NYC). The HIV Epidemiology & Field Services Program at the NYC DOHMH is authorized by the NYS Department of Health to conduct HIV/AIDS surveillance in NYC. Diagnostic providers are required by law to report all new diagnoses of HIV and AIDS and all laboratories are required to report positive HIV antibody tests (e.g., Western Blot, Immunofluorescence Assay, Multispot) as well as all viral loads, CD4 counts, and genetic resistance profiles. The Data Support Unit in the HIV Epidemiology and Field Services Program will assist with the matching of STD/Detox patient information with registry data. Lab data are prepared for import into the HIV registry through a rigorous process that involves examining test information and values for missing or erroneous data elements, cleaning the data as needed, matching them against existing case records, and standardizing key variables.

Because laboratories report patient viral loads over time and because NYC DOHMH can identify each case across time, it is possible to treat the surveillance data as an open cohort that individuals enter when they are first diagnosed with HIV. For example, we will use these data to quantify the time interval between individuals’ HIV diagnosis date and first viral load test as a marker for HIV care initiation, as well as the interval between diagnosis date and viral suppression/durable suppression.

The HIV Surveillance Registry includes: date of birth; sex; race/ethnicity; HIV transmission risk category; ZIP code of residence at time of HIV/AIDS diagnosis; concurrent HIV/AIDS diagnosis (AIDS diagnosis within 31 days of HIV diagnosis); vital status; viral load; and CD4 count.

#### Outcomes and analysis

The primary outcome variable for both the STD/Detox and Surveillance cohorts is DVS within 12 months of diagnosis, which will be calculated based on viral load values in the HIV Surveillance Registry. Persons with DVS will have: 1) at least two suppressed viral loads (≤200 copies/mL) within the first 12 months that are at least 90 days apart; 2) no unsuppressed intervening viral loads; 3) no unsuppressed viral load tests in the interval between achievement of DVS and the end of the 12 month period. The date of DVS will be based on the second suppressed viral load test that qualifies that person as having DVS.

For the STD/Detox cohort, the DVS outcome will be examined in two ways: more conservatively via a test of significant improvement and a priori increase of ≥5 %, and less conservatively to determine whether trends are increasing. The first hypothesis is that the proportion of subjects achieving DVS at each cohort year is different than 52 % (the most recent DVS estimate at the time of study development [27]). The hypothesis will be evaluated each year using two-sided proportion z-test. With a sample size of 75 for each cohort, we have at least 80 % power to detect viral suppression of 69 % using a 2-sided hypothesis test with level of significance of 5 %. Thus, the universal ART policy will be considered to have contributed to improved DVS if we reject the null hypothesis, because the sample durable suppression proportion is significantly greater than 52 %. Improvement will be considered inconclusive when the conclusion of the hypothesis test is ‘not having enough evidence to reject null hypothesis’. It is possible that viral suppression could worsen; this would be indicated if we reject the null hypothesis and DVS in the sample proportion is less than 52 %.

The second primary hypothesis is that the proportion of subjects from the Surveillance cohort that achieve DVS in each yearly cohort is different than 52 %. Because this cohort includes the entire population of newly HIV diagnosed cases in NYC, the proportion of subjects achieving DVS is considered a population proportion and sampling statistical techniques are not applicable. Therefore, an increase in population proportion of subjects achieving suppression of ≥5 % will be considered epidemiologically meaningful. Finally, the third hypothesis associated with the primary aims will assess the increasing trend of the proportion of subjects achieving DVS over the course of the study. The hypothesis is that there will be a significant positive trend in the proportion of subjects in the STD/Detox sample achieving DVS across 4 years of the study. This aim will be analyzed using the Cochran-Armitage trend test, which is less conservative than the analysis proposed to test the two previous hypotheses and thus, has a larger chance of correctly detecting successful policies. With 300 subjects, we have 80 % power to detect an overall increase of 12 % from the current 52 % (i.e., roughly 3 % every year).

#### Secondary outcomes

Secondary patient-level outcomes with the STD/Detox cohort will follow other HIV treatment cascade milestones: (1) Linkage to care (time to initial visit), (2) ART initiation (time to initiation of ART), (3) HIV treatment retention (sustained engagement of regular care defined as 1 or more primary care visits in 6 months, and (4) ART adherence (self-report). Additional outcomes include quality of life, sexual risk, and drug use risk. Additional covariate and predictor data is also being collected (see Table 1). Hypotheses 1 and 3 above will also be examined with the Surveillance cohort.

#### Qualitative in-depth interviews

At the 6-month follow-up visit, STD/Detox cohort participants will also complete a 30–45 min qualitative interview (*n* = 60). Qualitative assessment will elicit rich descriptions of participants’ experiences and motives in their own terms and language and provide complementary and elaborative data [59, 60]. Major themes will be compared and contrasted across cases over time and data will be analyzed as they are generated. If theoretical saturation is achieved for key themes before completing interviews, qualitative interviews will be stopped. Re-allocation of resources to complete additional qualitative interviews is an option should unanticipated events generate new questions.

### Meso-system: HIV treatment providers and primary care organizations

HIV primary care providers (*N* = 60) practicing in NYC will be recruited to provide information on adoption and implementation of HIV treatment policies and the impact of Affordable Care Act policy changes. Eligible providers will 1) work as a primary HIV medical care provider (i.e., physician, nurse practitioner, or physician’s assistant) with at least one HIV/AIDS patient on caseload (assessed at screening), and 2) be able to speak/understand English. Providers are initially identified through three publicly available lists (HIV Medicine Association [61], American Academy of HIV Medicine [62], and the New York State AIDS Institute [63]), randomly selected, and then matched as recent reporting providers in the NYC HIV surveillance system (ordered a viral load test in 2013 or 2014 that was reported to the NYC DOHMH). Providers will be interviewed in two cross-sectional cohorts covering Years 2-3 and Years 4-5 of the project. Providers are contacted by phone and given a description of the study, invited to participate, and, if interested, scheduled for a study visit. At the scheduled meeting, written informed consent is obtained prior to completing any research assessments.

Providers complete a brief survey and in-depth interview which will take approximately 60–90 min. Table 1 displays a list of indicator domains and variables to be assessed. Quantitative survey items include: personal and professional demographics, program characteristics, organizational level attitudes and beliefs (environmental uncertainty, change-related commitment, decision-making, stress, innovativeness, autonomy, information emphasis), and patient attitudes (patients with substance use disorders, patient-practitioner orientation). The qualitative interview covers domains including: understanding of universal ART policy, process of working with newly diagnosed HIV-infected patients, role of substance use and mental health in ART initiation, challenges and facilitators to providing medical care to HIV-infected patients, challenging patient conversations, impact of the Affordable Care Act, role of NYC and New York State in HIV primary care practice, and attitudes and practice with pre-exposure prophylaxis (PrEP). Questions about PrEP were included as it is an important form of HIV transmission prevention for high risk populations including negative partners of PLWH. For provider-level data collection, the dimensions Adoption, Implementation, and Maintenance will be assessed (see Table 1). Provider data will be used to elucidate information regarding Adoption, Implementation and Maintenance of ART guidelines and explore: barriers and facilitators to ART initiation, retention, adherence, and viral load suppression; and changes in practice and attitudes over time.

### Exo-system: neighborhood analysis

The purpose of the geospatial analyses is to examine the relationships of UHF-level exposures (specifically, socioeconomic conditions, social disorder, social cohesion, and spatial access to healthcare) to the key outcomes among the HIV Surveillance Registry cohort and patient-level STD/Detox cohort. Key outcomes include DVS, viral suppression, and other indicators along the HIV treatment cascade (linkage to care, ART initiation, and HIV care retention). These outcomes will also be explored as a function of HIV transmission category and among racial/ethnic subgroups.

#### Data sources

Existing administrative databases will be analyzed to describe UHF-level socioeconomic conditions (e.g., poverty rates, education); social disorder (e.g., alcohol outlet density); social cohesion (e.g., percent housing units leased); and spatial access to HIV-related services (e.g., HIV primary care). These environmental dimensions will be ascertained using methods based on the investigator’s prior work [64–68]. For both the STD/Detox cohort and for the entire HIV Surveillance Registry cohort, ZIP code of residence at HIV diagnosis will be obtained and linked to the patients' UHF district. Each UHF district consists of 3–9 adjacent zip codes that have similar sociodemographic characteristics. The NYC DOHMH uses these districts to track and analyze local patterns of health outcomes and healthcare service delivery. Median district population size is approximately 195,000 (range: 31,000–477,500). Similar to the comprehensive STD/Detox cohorts, Reach, Efficacy and Maintenance will be assessed using all new diagnoses from the NYC DOHMH HIV Surveillance Registry (see Table 1).

#### Analysis

Relationships between UHF characteristics and the outcomes (i.e., viral suppression and DVS within 12 months of diagnosis) will be investigated using exploratory data analysis and multilevel modeling [69, 70]. Individuals will be nested within UHF districts, and UHF level predictors will be used to examine outcomes at the individual level. The collection of extensive data on all subjects in the STD/Detox cohort will allow for more comprehensive examination. Hierarchical regression will be used to explore significant differences between participants who achieve viral suppression and those who do not in both the HIV Surveillance Registry cohort and STD/Detox cohort to identify what factors influence differences. Such factors would potentially support reallocation of resources to increase rates of viral suppression in NYC. If the relationships between place characteristics and outcomes in the STD/Detox cohort data are similar to those found in the Surveillance cohort, we will have greater confidence that the relationships found among the smaller STD/Detox cohort are true.

### Exo/macro-system: policy

To document policy adaptation or changes that pertain to NYC, materials related to HIV testing, treatment, and prevention guidelines will be collected at the city, state and national level. These materials will include, but are not limited to, modifications to existing guidelines, committee meeting minutes, and funding decisions. Importantly, upcoming changes related to Affordable Care Act implementation, including the role of Health Homes for people living with HIV/AIDS, insurance exchanges, Medicaid expansion, and the AIDS Drug Assistance Program will be documented and assessed at the client, provider and system levels. Insurance information (and other payer data) will be collected from participants; information will also be collected from providers about reimbursement methods and how these impact patient outcomes. Policies will be archived by date to link with changes in client and provider level data.

### Collaborative board

The Collaborative Board will provide the primary basis of collaborative action for grant implementation, review of ongoing data and analysis, and feedback and recommendations on policy and practice guidelines related to the HIV treatment cascade in NYC. The Board is comprised of HIV treatment providers, clinical researchers, consumers living with HIV, and NYC DOHMH representatives from the HIV Epidemiology and Field Services Program, Bureau of STD Control, and the HIV Care and Treatment Program. Study investigators will convene the board, present project data updates, and facilitate discussion. The investigative team and NYC DOHMH staff will not be present during deliberation related to specific recommendations, so that decisions remain independent of the other research processes. However, including all key stakeholders in dialogue is essential for understanding the context of policy implementation. Board meetings will be audio recorded for analytic purposes (except recommendation deliberations); recommendations will be collected and documented. The Board will meet approximately twice per year.

## Discussion

This study protocol is being implemented in the context of major shifts in the local HIV epidemic, including decreasing HIV diagnosis rates. The changing context has also resulted in a reframing of state policy. In June 2014, New York State Governor Andrew Cuomo announced a plan to effectively “End the HIV Epidemic” by reducing the HIV incidence rate to below epidemic levels - from approximately 3000 cases per year to 750 cases by 2020 [71]. The plan proposes to address the following three objectives: (1) identify persons with HIV and link them to health care; (2) retain persons in health care and get them on ART to maximize viral suppression; and (3) provide access to PrEP for high-risk persons. Following this press release, the Governor appointed a Task Force to develop a set of recommendations for achieving these three objectives. The Task Force convened October 2014 through January 2015 and was comprised of over 60 community, advocacy, and academic/research experts, including Drs. Des Jarlais, Remien, and Urbina, all involved in the current project (a list of Task Force members is available here: http://www.health.ny.gov/diseases/aids/ending_the_epidemic/docs/members.pdf). The final “blueprint” recommendations from the Task Force were approved on January 13, 2015 and were released to the public in April 2015. Recommendations come from four subcommittees: care, data, housing and supportive services, and prevention. The recommendation domains are complementary to data collected in the AFA Study.

The ambitious New York State goal of “Ending the Epidemic” relies in part on improvements in rates of viral load suppression. The proposed research: (1) studies a policy (universal ART) clearly aimed at ending the epidemic; (2) utilizes a mixed method design and multi-system data sources to evaluate the local success of the policy and provide a broader and more contextual understanding of PLWH treatment experiences; (3) examines neighborhood characteristics (e.g., poverty rates, social cohesion, spatial access to HIV care) that may affect the likelihood of reaching viral suppression and how place characteristics could generate racial/ethnic disparities in HIV infection; (4) assesses the policy concurrent to service changes brought about by the Affordable Care Act; and (5) in partnership with the NYC DOHMH and collaborative board members, will disseminate findings in real time throughout the funding period in the context of a rapidly changing environment. The innovative components of this project should help to produce critical public health information with broad reach.

### Collaborative board recommendations

The changing context and encouraging shifts in the NYC HIV epidemic suggest the need to expand the focus of the AFA Study. At a discussion of the Collaborative Board in January 2015, there was consensus to expand the objectives of the study to focus equal time on facilitators of linkage to care, retention in care, initiation of ART, and ultimately viral suppression. In line with this shift in emphasis on what is working well, the Collaborative Board also agreed with the plan to begin enrolling any persons with HIV, in addition to newly diagnosed and out of care persons with HIV. In this way, those who are HIV positive and in care can share information on what has facilitated and supported their successful HIV care trajectories.

### Limitations

Several limitations are worth noting in the design and implementation of the study. First, although the NYC DOHMH HIV Surveillance Registry is one of the best in the nation, there are data reporting and processing lags that affect the timeliness of viral load suppression analyses. An additional limitation to surveillance data is the difficulty in identifying out-migration of HIV positive individuals who have moved away from the city. A third limitation is the difficulty, already noted, in recruiting newly HIV-diagnosed individuals among a reduced number of HIV diagnoses, as well as the challenge for patients to manage their new diagnosis and feel prepared to participate in research at the same time. This recruitment challenge could lead to a higher likelihood of non-representative samples. Fourth, there is no consistent definition of DVS in use; the definition proposed in the AFA Study may differ from other reports. Finally, because neighborhood level data are based on previously existing data sources, there may be placed-based constructs that are important but are unmeasurable because data are unavailable.

### Study status

The AFA Study received NIH funding in July 2013 and will continue through March 2018. The first patient-level STD/Detox cohort was recruited in 2014 and Cohort 2 (January-December 2015) recruitment is underway. Recruitment of the first of two cross-sectional cohorts of providers is also underway. Initial analysis and manuscript development is underway to examine the proportion of newly diagnosed individuals (2009–2012) who reach viral load suppression and DVS within 12 months of diagnosis. This will be an analysis of geographic (UHF-level) predictors of viral load suppression as a function of HIV transmission risk (i.e., injection drug use, heterosexual sexual transmission, or male to male sexual transmission).

## Abbreviations

AFA, ART for all study; AIDS, acquired immune deficiency syndrome; ART, antiretroviral treatment; DVS, durable viral suppression; HIV, human immunodeficiency virus; IRB, institutional review board; MSM, men who have sex with men; NIH, National Institutes of Health; NYC DOHMH, New York City Department of Health and Mental Hygiene; PLWH, people living with HIV; PrEP, pre-exposure prophylaxis; RE-AIM, reach, efficacy, adoption, implementation, and maintenance model; STD, sexually transmitted disease; STI, sexually transmitted infection; UHF, united hospital fund
